# Sarcoplasmic Phospholamban Protein Is Involved in the Mechanisms of Postresuscitation Myocardial Dysfunction and the Cardioprotective Effect of Nitrite during Resuscitation

**DOI:** 10.1371/journal.pone.0082552

**Published:** 2013-12-30

**Authors:** Yu Huang, Qing He, Lei Zhan, Min Yang

**Affiliations:** 1 The Third People's Hospital of Chengdu, The Second Affiliated Hospital of Chengdu, Chongqing Medical University, Chengdu, China; 2 Department of Respiratory Disease, West China Hospital of Sichuan University, Chengdu, China; 3 Department of Intensive Care Unit, The Second Hospital of Anhui Medical University, Anhui, China; Loyola University Chicago, United States of America

## Abstract

**Objectives:**

Sarcoplasmic reticulum (SR) Ca^2+^-handling proteins play an important role in myocardial dysfunction after acute ischemia/reperfusion injury. We hypothesized that nitrite would improve postresuscitation myocardial dysfunction by increasing nitric oxide (NO) generation and that the mechanism of this protection is related to the modulation of SR Ca^2+^-handling proteins.

**Methods:**

We conducted a randomized prospective animal study using male Sprague-Dawley rats. Cardiac arrest was induced by intravenous bolus of potassium chloride (40 µg/g). Nitrite (1.2 nmol/g) or placebo was administered when chest compression was started. No cardiac arrest was induced in the sham group. Hemodynamic parameters were monitored invasively for 90 minutes after the return of spontaneous circulation (ROSC). Echocardiogram was performed to evaluate cardiac function. Myocardial samples were harvested 5 minutes and 1 hour after ROSC.

**Results:**

Myocardial function was significantly impaired in the nitrite and placebo groups after resuscitation, whereas cardiac function (i.e., ejection fraction and fractional shortening) was significantly greater in the nitrite group than in the placebo group. Nitrite administration increased the level of nitric oxide in the myocardium 5 min after resuscitation compared to the other two groups. The levels of phosphorylated phospholamban (PLB) were decreased after resuscitation, and nitrite increased the phosphorylation of phospholamban compared to the placebo. No significant differences were found in the expression of sarcoplasmic reticulum Ca^2+^ ATPase (SERCA2a) and ryanodine receptors (RyRs).

**Conclusions:**

postresuscitation myocardial dysfunction is associated with the impairment of PLB phosphorylation. Nitrite administered during resuscitation improves postresuscitation myocardial dysfunction by preserving phosphorylated PLB protein during resuscitation.

## Introduction

According to recent epidemiological studies, approximately 70% of cardiac arrest (CA) patients who had restoration of spontaneous circulation (ROSC) died before hospital discharge [1]–[3]. Postresuscitation myocardial dysfunction is one of the major factors contributing to the high mortality after initial resuscitation [4]. Although cardiopulmonary resuscitation (CPR) strategies are continually updated, the mechanism of postresuscitation myocardial dysfunction remains poorly understood, and the available data are very limited. Novel therapies that might improve postresuscitation myocardial dysfunction require further exploration.

Cardiac sarcoplasmic reticulum (SR) plays a central role in excitation-contraction coupling and myocardial contractile dysfunction through its Ca^2+^-modulating function [5]. The Ca^2+^ modulation of SR is associated with Ca^2+^-handling proteins, including sarcoplasmic reticulum Ca^2+^ ATPase (SERCA2a), phospholamban (PLB) and ryanodine receptors (RyRs). Previous studies demonstrated that the ischemia-reperfusion (I/R) process might induce down-regulation of the expression and function of the SR proteins, which are involved in myocardial dysfunction after I/R injury [6], [7]. Furthermore, impairment of SR Ca^2+^ handling was shown to be involved in the Ca^2+^ overload observed during I/R injury, which induces hypercontraction of myofibrils and further mitochondrial injury in a Ca^2+^-related manner [8], [9]. Such alterations might finally contribute to myocardial reperfusion injury and thus compromise cardiac contractility. Recent studies have shown that SR Ca^2+^-handling proteins might be essential targets for protection against acute myocardial I/R injury [10].

Nitric oxide (NO) is an important regulator of myocardial function; it protects myocardium against I/R injury by preserving SR Ca^2+^-handling proteins via related pathways such as the protein kinase G pathway [11]–[14]. Nitrite is an important donor for NO generation via NOS-independent reduction especially during hypoxia, acidosis or the I/R process by iron-containing enzymes such as deoxyhemoglobin and myoglobin [15]. Recent studies showed that the administration of nitrite might attenuate I/R injury in the heart, brain or kidney [16], [17]. Because CA/CPR is an acute global I/R process, we hypothesize that postresuscitation myocardial dysfunction is related to the impairment of SR Ca^2+^-handling proteins and that nitrite might improve postresuscitation myocardial dysfunction by a mechanism involving the modulation of SR Ca^2+^-handling proteins.

## Materials and Methods

This study was approved by the Institutional Animal Care and Use Committee of the West China Medical Center of Sichuan University (Permit Number: 2011-12). The investigation conformed to National Institutes of Health guidelines for ethical animal research.

### Animal preparatory phase

Male Sprague-Dawley rats weighing 350–450 g were fasted overnight with free access to water. The rats were anesthetized with 45 mg/kg of pentobarbital delivered by intraperitoneal injection. An additional dose (10 mg/kg) was administered at an interval of approximately 1 hour to maintain anesthesia. The trachea was then intubated with a 14-gauge cannula, and mechanical ventilation was delivered at 100 breaths per min with a tidal volume of 0.65 ml/100 g and a fraction of inspired oxygen (FiO2) of 0.21 (HX-100E, TME Technology Co, Ltd, Chengdu, China). A heparin-filled (140 U/L) 20G PE50 catheter was inserted through the left carotid artery for the measurement of arterial blood pressure and blood sampling. A 20G PE50 catheter was advanced through the right carotid artery into the left ventricle, for monitoring the waveform of left ventricular pressure (LVP). Another PE50 catheter was advanced through the left jugular vein into the right atrium for measuring atrial pressure. All of the catheters were connected to a high sensitivity pressure transducer (PT-100, TME Technology Co, Ltd, Chengdu, China). Electrocardiograph lead II was recorded continuously. A 24G PE50 catheter was inserted into the left femoral vein for fluid and drug administration. The body temperature was maintained at 37°C (±0.5°C) with a heat lamp.

### CA/CPR model

We performed a standard CA model with the goal of nearly 100% resuscitation, as previously described [18], [19]. CA was induced by bolus administration of 40 µg/g of potassium chloride, which shows no damage to the myocardium but is quickly eliminated from the plasma [18], [19]. Mechanical ventilation was discontinued simultaneously. CA was confirmed by a mean arterial pressure (MAP) less than 20 mmHg and asystole on ECG. After 9 min of untreated CA, ventilation was resumed, FiO_2_ was increased to 1.0 and manual chest compression was started synchronously with ventilation. Chest compression was performed at a rate of 200/min and a depth of nearly one half of the thoracic anteroposterior diameter, with equal compression-relaxation periods. At the initiation of chest compression, the rats received nitrite sodium (1.2 nmol/g, a dose determined in previous studies) or placebo (0.9% saline of equal volume) [20]. The drugs were delivered in a randomized manner by the sealed envelope method. All investigators performing CPR and interpreting the outcome assessments were blinded to the medication.

ROSC was defined as a sustained MAP of >60 mmHg [21]. If no ROSC was achieved after 6 min of continuous compression, CPR was terminated. No CA was induced for rats in the sham group. Mice subjected to the sham operation that were not subjected to cardiac arrest were used as controls. Ringer's lactate solution was continuously infused at a rate of 3 ml/h during the monitoring phase.

### Monitoring and Measurements

Rats that achieved ROSC were monitored for 90 min after resuscitation. Trans-thoracic echocardiography of cardiac function was performed at baseline and during the 90-minute monitoring after resuscitation. After the chest was shaved, 2-dimensional and M-mode images were obtained with a Vivid 7 ultrasound machine (GE Medical Systems, Horten, Norway) utilizing an 11.5 MHz probe at the level of the papillary muscles. The ejection fraction and fractional shortening was calculated with the manufacturer's software.

The arterial blood pressure, right atrial pressure, LVP waveform and electrocardiogram during CPR were continuously monitored with a computer-based BL-420 biofunction experimental system (BL-420F, TME Technology Co, Ltd, Chengdu, China). Coronary perfusion pressure (CPP) during CPR was monitored (coronary perfusion pressure was assessed as arterial diastolic pressure minus right atrial pressure) with the same system. The maximal rate of LV pressure increase (dP/dtmax) and the maximal rate of LV pressure decline (−dP/dtmax) were additional measures of cardiac systolic and diastolic function, respectively.

### Western blot analysis of Ca^2+^-handling proteins

We analyzed the expression of SERCA2a, phosphorylated PLB and RyR by Western blot. The rats were euthanized by intraperitoneal injection of pentobarbital (150 mg/kg) 5 min after ROSC and 90 min after resuscitation, and myocardium obtained from the anterior wall of the left ventricle was snap-frozen at −70°C. Samples of myocardium were pulverized and homogenized with lysis buffer (Tris-HCl, pH 8.0, urea; with protease, phosphatase, and kinase inhibitors). After centrifugation at 700 g for 10 min, the supernatant was collected and then centrifuged at 14000 g for 30 min, and the final supernatant was collected. The proteins contained in the lysates were separated by SDS-PAGE. The separated proteins were then transferred electrophoretically onto PVDF sheets. The membranes were blocked in TBS solution with 5% nonfat dry milk for 1 h at room temperature.

The blots for each protein were incubated with a 1∶5000 dilution of mouse anti-SERCA2a antibody (ab2861, Abcam, Hong Kong), rabbit anti-PLB antibody (phospho- S16+T17, ab62170, Abcam, Hong Kong) or anti-RyR antibody (ab59225, Abcam, Hong Kong) at 4°C overnight and then incubated with peroxidase-conjugated goat anti-rabbit IgG (Sigma, St. Louis, MO, USA) at room temperature for 1 h. The protein signals were analyzed with the Quantity One system (Version 4, Bio-Rad Laboratories, Inc., Hercules, California). The expression of each protein was normalized to β-tubulin.

### Measurement of nitric oxide (NO) levels in myocardium and plasma

For measuring myocardial NO after CA/CPR, rats were euthanized by injection of pentobarbital 5 min after ROSC, and then myocardial samples were snap-frozen at −70°C. For measuring plasma NO, arterial blood sampling was performed 5 min after ROSC. The nitric oxide level in the cytoplasmic solution and plasma sample was determined by chemical colorimetric assay using the Nitric Oxide Assay Kit (S0023, Beoytime Institute of Biotechnology, China) according to the manufacturer's instructions [22].

**Figure 1:**
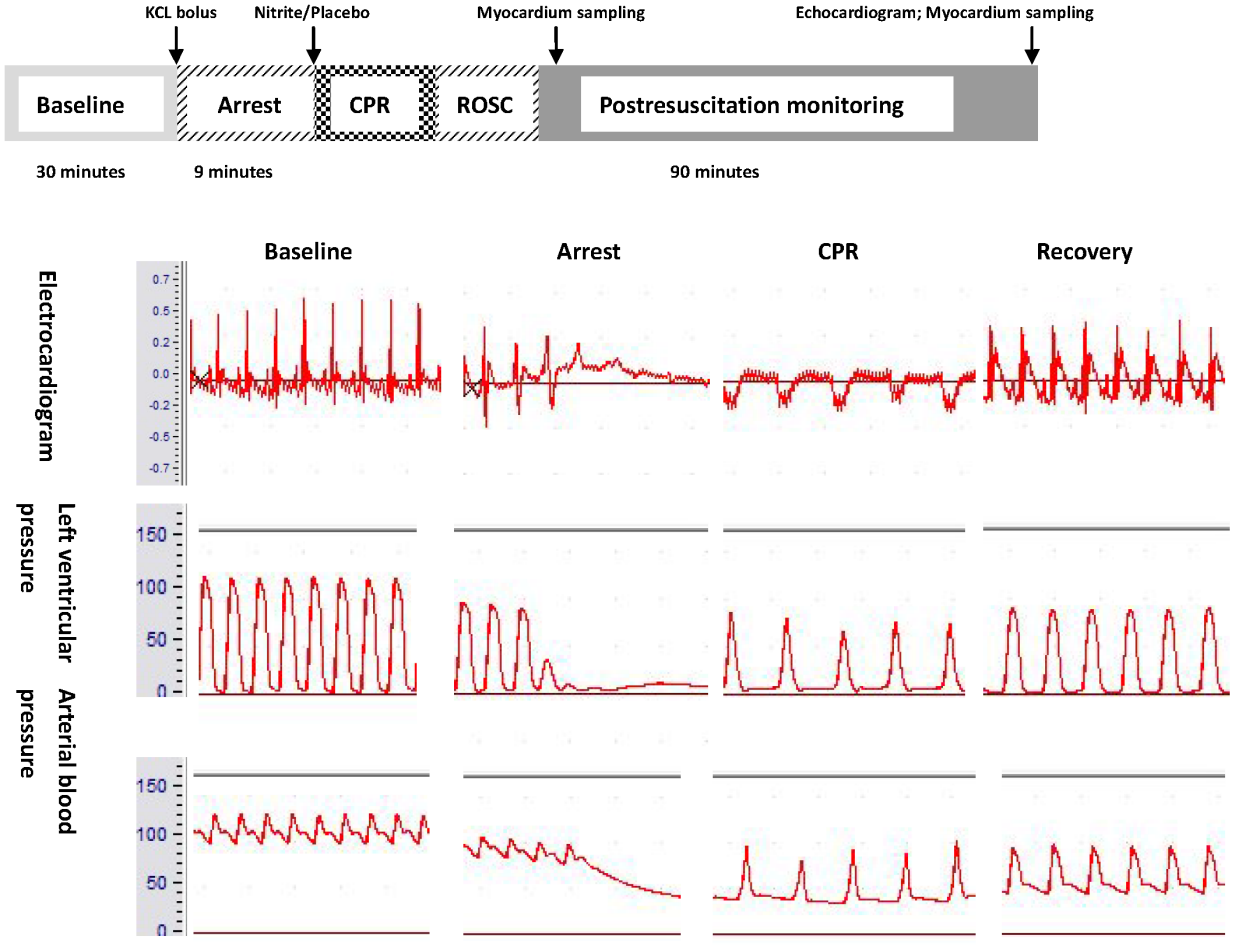
Experimental protocol.

The flow diagram of experimental protocol is shown in Figure 1.

### Statistical analyses

Measurements were reported as means ± SD. Statistical significance of differences of means of continuous variables between groups was determined with single-factor ANOVA with a Bonferroni post-hoc test when the variables were normally distributed. One-way ANOVA on ranks was used when the variables were not normally distributed. Comparisons between time-based measurements within each group were performed with repeated measurements analysis of variance. A 2-tailed Fischer's exact test was used for ROSC rates. A value of P<0.05 was considered significant.

## Results

There were no differences in any hemodynamic or respiratory parameters at baseline between the three groups (Table 1). The ROSC rates in the placebo and nitrite groups were similar. No significant difference in CPP during chest compression was found between the nitrite group and the placebo group. The CPR time in the nitrite group was insignificantly shorter than that of the placebo group (Table 2).

**Table 1:** Baseline characteristics of three groups.

	Sham group (n = 12)	Placebo group (n = 26)	Nitrite group (n = 27)
HR (beat/min)	380±29	370±38	374±36
MAP (mmHg)	132±14	130±25	138±22
dp/dtmax (mmHg/sec)	7588±275	7546±330	7599±266
−dp/dtmax (mmHg/sec)	−6696±220	−6527±376	−6657±310
EF (%)	82.2±3.7	81.1±3.3	82.1±2.8
FS (%)	47.5±4.5	45.7±3.5	47.2±3.4
pH	7.45±0.02	7.46±0.04	7.46±0.02
Pco_2_ (mmHg)	30±4	34±5	33±4
Po_2_ (mmHg)	115±25	120±28	122±20
Lactate (mmol/L)	0.33±0.05	0.38±0.11	0.37±0.07

HR: heart rate; MAP: mean arterial pressure; dp/dtmax: maximal rate of LV pressure increase; −dp/dtmax: maximal rate of LV pressure decline; EF: ejection fraction; FS: fractional shortening.

Values are means±SD.

**Table 2:** CPR characteristics and basic hemodynamic parameters after ROSC.

	Sham group	Placebo group	Nitrite group
ROSC (n)	NA/12	24/26	25/27
CPP during CPR (mmHg)	NA	25±3	25±3
Time of CPR (s)	NA	68±39	50±33
HR at 1h after ROSC (beat/min)	378±35	360±37	389±40
MAP at 1h after ROSC (mmHg)	131±25	117±37	125±34
VF/VT (n)	0	0	0

ROSC: return of spontaneous circulation; CPP: coronary perfusion pressure; CPR: cardiopulmonary resuscitation; HR: heart rate; MAP: mean arterial pressure; VF: ventricular fibrillation; VT: ventricular tachycardia.

Values are means±SD.

### Nitrite improved cardiac dysfunction after CA/CPR

**Figure 2:**
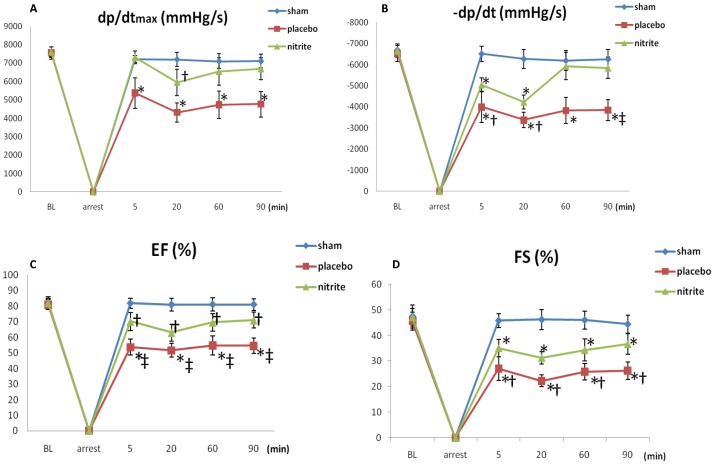
Postresuscitation myocardial function evaluated by invasive monitoring and echocardiography (n = 6 for sham group; n = 10 for placebo or nitrite group). **A**, The maximal rate of LV pressure increase (dp/dtmax) of each group, *P<0.01 vs. sham and nitrite groups; †*P*<0.05 vs. sham group. **B**, The maximal rate of LV pressure decline (−dp/dtmax) of each group, *P<0.01 vs. sham group; †*P*<0.05 vs. nitrite group; ^‡^ *P*<0.01 vs. nitrite group. **C**, the ejection fraction of each group, *P<0.01 vs. sham group; †*P*<0.05 vs. sham group; ^‡^ *P*<0.01 vs. nitrite group; **D**, the fractional shortening of each group; *P<0.01 vs. sham and nitrite groups; †*P*<0.05 vs. nitrite group.

At 90 min after ROSC, the heart rate and MAP in placebo and nitrite group showed no significant differences compared to sham group. The HR and MAP in the nitrite group tended to be higher than in the placebo group, but the differences were not statistically significant. No ventricular tachycardia or ventricular defibrillation occurred in either group after ROSC (Table 2). In cardiac functional evaluation by invasive monitoring, the dP/dtmax and −dP/dtmax (absolute values) in the nitrite and placebo groups were both decreased after ROSC. In the nitrite group, the dP/dtmax and −dP/dtmax were significantly higher than those of the placebo group during the 90 minutes after ROSC (Figure 2).

The echocardiographic evaluations performed 30 min, 60 min, and 90 min after ROSC revealed that the ejection fraction and fractional shortening in the nitrite and placebo groups were both decreased compared to the sham group. Furthermore, the ejection fraction and fractional shortening in the nitrite group were significantly higher compared to the placebo group (Figure 2). Thus, cardiac dysfunction was induced by the CA/CPR process, and nitrite administered during CPR improved cardiac dysfunction after resuscitation.

### The measurement of NO in myocardium

**Figure 3:**
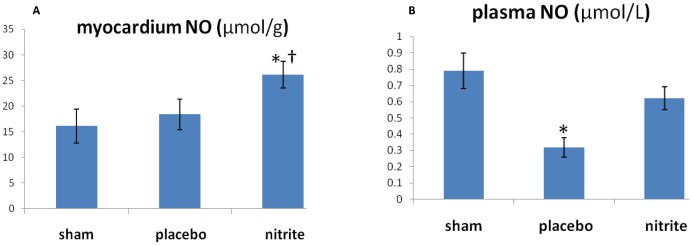
Nitric oxide (NO) levels after cardiac arrest and resuscitation. **A**, The measurement of NO in myocardium (n = 6 for sham group; n = 10 for placebo or nitrite group). Nitrite administration significantly increased the NO level after resuscitation. *P<0.01 vs. sham group; †*P*<0.05 vs. placebo group. **B**, The measurement of NO in plasma (n = 6 for sham group; n = 10 for placebo or nitrite group). *P<0.01 vs. sham group and nitrite group.

Myocardium and plasma NO levels 5 min after ROSC were analyzed. The myocardial NO level in the placebo group tended to be higher than in the sham group, and in the nitrite group, the NO level was significantly higher than in the other two groups (Figure 3A). In the comparison of plasma NO, the NO level in the placebo group decreased significantly after ROSC compared with the sham group. The plasma NO level in the nitrite group also decreased after ROSC, but it was significantly higher than that of placebo group (Figure 3B).

### The preservative effect of nitrite on SR Ca^2+^-handling proteins in the CA/CPR model

**Figure 4:**
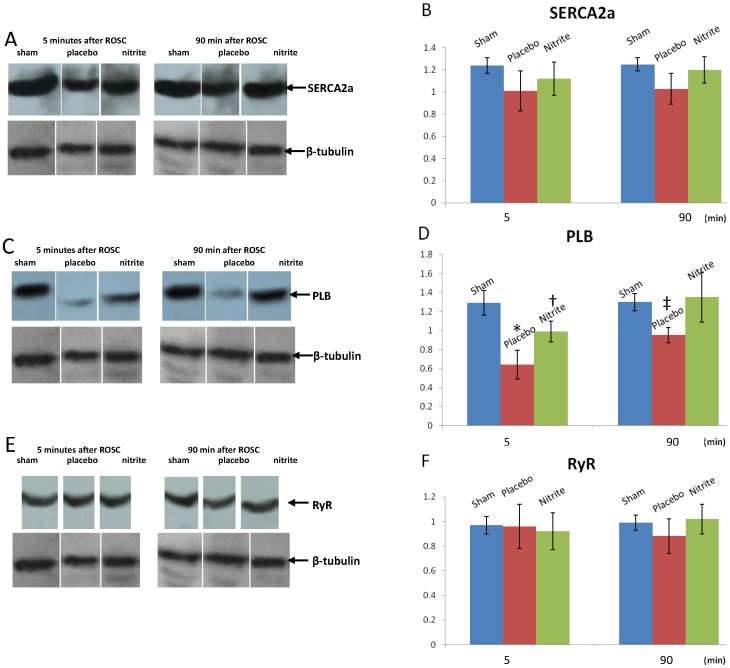
Representative Western blot images of SERCA2a, PLB and RyR 5(n = 6 for sham group; n = 10 for placebo or nitrite group) and 90 min (n = 6 for sham group; n = 10 for placebo or nitrite group) after ROSC are shown in A, C, E. The level of phosphorylated PLB was decreased significantly after resuscitation in the placebo group and nitrite administration preserved phosphorylated PLB during resuscitation. *P<0.01 vs. sham group; †*P*<0.05 vs. sham and placebo groups; ‡*P*<0.05 vs. sham and nitrite groups.

We investigated the association between the effects of nitrite and the key proteins related to SR Ca^2+^ handling. We compared the expression levels of proteins related to Ca^2+^ uptake in the SR, including SERCA2a and PLB. Five min after ROSC, the expression levels of phosphorylated PLB in the nitrite and placebo groups were both decreased significantly compared to the sham group, and this reduction was attenuated in the nitrite group. The level of phosphorylated PLB remained lower in the placebo group compared to the sham group 90 min after resuscitation, and no significant difference was found between the nitrite group and the sham group (Figure 4C, D). However, there were no significant differences in the expression level of SERCA2a between these groups either 5 min after ROSC or 90 min after resuscitation (Figure 4A, B). In the comparison of the expression levels of RyR, a protein related to Ca^2+^ release, no significant difference was found at either time point (Figure 4E, F).

## Discussion

The abnormality of SR Ca^2+^-handling proteins plays an essential role in myocardial injury and myocardial dysfunction after the I/R process. SERCA2a and RyR are proteins which directly attribute to the Ca2+ uptake and Ca2+ release of SR respectively. PLB is a major modulator of SERCA2a activity, phosphorylation of PLB can increase the SERCA2a activity while dephosphorylated PLB act as an inhibitor of SERCA2a activity [23]. SERCA2a, RyR and PLB are fundamental proteins which regulate SR Ca^2+^-handling. Our findings suggest that, in this CA/CPR model, postresuscitation myocardial dysfunction is mainly associated with altered PLB, while SERCA2a and RyR are not involved. Furthermore, nitrite administration may improve postresuscitation myocardial dysfunction, and the preservation of phosphorylated PLB is involved in its mechanism.

SERCA2a and PLB play an important role in myocardial contractility and intracellular Ca^2+^ homeostasis by regulating the Ca^2+^ uptake function. Previous studies showed that reductions of SERCA2a and phosphorylated PLB were closely related to myocardial dysfunction induced by I/R injury [6], [7]. Prunier and colleagues also showed that upregulation of SERCA2a might protect myocardium, prevent ventricular arrhythmias and reduce areas of infarct after I/R by modulating Ca^2+^ overload [24] and that phosphorylation of PLB causes an increase in SERCA2a activity [23]. Likewise, in our study, myocardial function was significantly depressed after CA/CPR, and phosphorylated PLB was significantly decreased after resuscitation in the placebo compared to the sham group. It is conceivable that the impairment of PLB phosphorylation is involved in the stunning-like myocardial dysfunction after resuscitation. However, although there was a similar trend with SERCA2a, but it did not reach statistical significance. Also we found no significant alteration in the expression RyR. Thus, we suggested that in this CA/CPR model, postresuscitation myocardial dysfunction is mainly associated with the alteration of PLB phosphorylation, but not the expression of SERCA2a and RyR. This differs from the myocardial dysfunction secondary to other I/R models, such as coronary diseases and revascularization after myocardial infarction. CA/CPR process is an acute I/R process with short-term ischemia, and we observe a maximal decrease of the proteins at 5 minutes of ROSC, which is very fast. So we suggest that it is reasonable that the myocardial dysfunction is more likely related to phosphorylation of PLB rather than expression of SERCA2a. Kim et al. suggested that myocardial stunning secondary to an acute I/R process was associated with impaired Ca^2+^ handling, in which dysfunction of SERCA2a subsequent to a decrease of PLB phosphorylation was involved [25]. These findings were consistent with ours.

Specifically, in acute myocardial I/R models, cytosolic Ca^2+^ overload induced by impaired SR Ca^2+^ handling in the first minutes of reperfusion (i.e., 5–10 min) might be important in the mechanism of myocardial dysfunction [8], [10], [26]. The oscillatory elevation of cytosolic Ca^2+^ may cause mitochondrial injury in a mitochondrial Ca^2+^ uniporter-related manner [8], [27], which subsequently induces abnormal energy metabolism and reactive oxygen species (ROS) generation. In addition, a recent study utilizing an intact heart model showed that, at the onset of reperfusion, the depletion of SR Ca^2+^ content and corresponding increase of cytosolic Ca^2+^ played an important role in myocardial stunning after acute I/R [28]. Such an abnormality would reverse in 5–10 minutes after reperfusion. Importantly, Abdallah and colleagues have found that these impairments are closely associated with the phosphorylation of PLB [26], [29]. In our study, it was shown that at 5 min after resuscitation, phosphorylated PLB was significantly decreased in the placebo compared to sham group. Thus, considering that CA/CPR is an acute global I/R process and causes postresuscitation myocardial stunning, it is conceivable that a decrease of PLB phosphorylation in the first minutes of reperfusion might play an important role in the postresuscitation myocardial dysfunction.

The second important finding of our investigation is that the preservation of phosphorylated PLB might be involved in the protective effect of nitrite and NO on postresuscitation myocardial function.

NO has been reported to mediate cytoprotection in animal models of I/R injury [16], [17]. In a CA/CPR model, it has recently been shown that improving NO formation would protect cardiac function after resuscitation [30]–[32]. Although recent studies have also shown that the cardioprotective effect of NO during resuscitation might be related to the attenuation of CA-induced mitochondria injury and ROS generation [30], [31], whether SR Ca^2+^-handling functions or SR Ca^2+^-handling proteins are involved in the mechanism is not clear.

In the present study, a decrease of plasma NO and an increase of myocardial NO were found in the placebo group 5 min after resuscitation compared to the sham group. This finding indicated that an increase of myocardial NO generation might be induced by the I/R process; however, the increase was limited by the depletion of substrates in the plasma, and also the inhibition of NOS activity under hypoxic conditions [33]–[35]. Therefore, we used intravenous nitrite as a NO supplement treatment, and in the nitrite group, NO generation was significantly increased in both the plasma and the myocardium because of the exogenous nitrite supplement, which served as a donor for NO generation.

Our results showed that nitrite significantly preserved cardiac function compared to saline, accompanied by an increased myocardial NO level, and synchronous results were found in the comparison of phosphorylated PLB. These results suggest that the cardioprotective effect of NO during CA/CPR is associated with the upregulation of phosphorylated PLB protein and the SR Ca^2+^ uptake function. Recent studies have demonstrated that NO acts as an important regulator of SR function mainly by modulating the phosphorylation of PLB. In vitro, Kasseckert et al. have found that stimulation of cGMP might activate cGMP-dependent protein kinase (PKG) and thereby increase the phosphorylation of phospholamban and modulate Ca^2+^ uptake during reperfusion [36]. Abdallah and colleagues have also found that NO conferred myocardial protection against acute I/R injury, which was related to the upregulation of phosphorylated PLB via the cGMP pathway in the early stage of reperfusion [26], [29]. Thus, it has been shown that NO might regulate PLB phosphorylation via the guanylyl cyclase/cGMP/PKG pathway. In vivo, Jung et al. have found that nitrite increased the cGMP level via a NO/guanylyl cyclase/cGMP pathway during I/R [16]. In a CA/CPR model, Minamishima and colleagues have found that NO inhalation might induce a cytoprotection effect and affect postresuscitation outcomes via guanylate cyclase-dependent mechanisms [37]. Therefore, we suggest that, in our model, the cardioprotective effect of NO during CA/CPR is related to the modulation of PLB phosphorylation via a guanylyl cyclase/cGMP-dependent mechanism.

In addition, intravenous nitrite significantly increased the NO level in myocardium early after CPR and thereby induced an increase in PLB phosphorylation and postresuscitation myocardial function, suggesting that the early stage of reperfusion is a window of opportunity for modulating SR Ca^2+^ handling and attenuating myocardial injury during CPR. Our results also showed that the level of phosphorylated PLB tended to be reversed in the nitrite group 90 min after ROSC, which again suggests that myocardial stunning after CPR is mainly associated with reversible mechanisms and can be improved by appropriate therapies.

A limitation of this study is that we focused only on the expression and phosphorylation of SR Ca^2+^-handling proteins. Also, PLB is phosphorylated at residues Ser-16 and Thr-17 with different mechanisms (including PKA and CaMKII pathways), and we only examined total phosphorylated PLB. Therefore, we anticipate further mechanistic studies including the PLB phosphorylation at ser-16 and Thr-17, we also anticipate further studies including the alteration of the guanylyl cyclase/cGMP pathway and the possible involvement of other pathways related to NO or SR Ca^2+^ handling, such as Akt- and AMP-activated protein kinase and cAMP-dependent protein kinase pathways to better understand and confirm the role of SR proteins and related pathways in CA/CPR.

Our findings have shown that the impairment of PLB, an important SR Ca^2+^ uptake protein, is involved in the mechanism of postresuscitation myocardial dysfunction and that the cardioprotective effect of nitrite during resuscitation is also associated with the modulation of PLB phosphorylation. Recently, different exogenous NO therapies, such as intravenous nitrite or sodium nitroprusside administration and NO inhalation, have been shown to be beneficial in resuscitation [31], [37], [38]. Our study provides further evidence for the mechanisms of nitrite-induced myocardial protection in resuscitation, and the molecular mechanisms and clinical effects of these therapies require further study.
